# Supplementary material for: Perspectives on Corrosion Inhibition Features of Novel Synthesized Gemini-Fluorinated Cationic Surfactants Bearing Varied Spacers for Acid Pickling of X60-Steel: Practical, and In Silico Calculations
Source: Materials (Basel). 2023 Jul 24;16(14):5192. doi: 10.3390/ma16145192 (PMC10383753; doi:10.3390/ma16145192)
Supplement: Supplementary file 1 [file materials-16-05192-s001.zip › materials-2423051-supplementary.pdf]

Supporting information

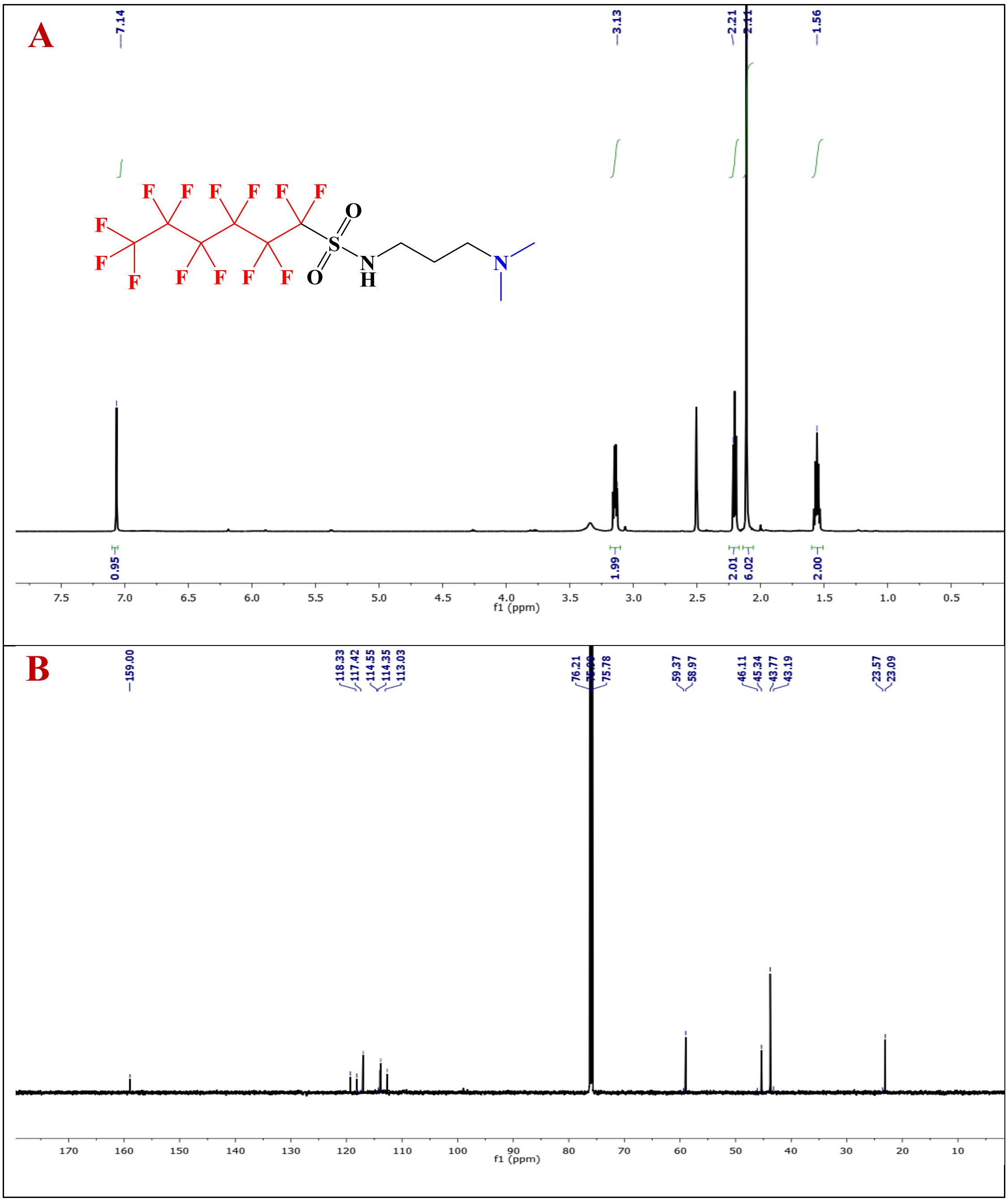
A
7.14
3.13
2.21
2.11
1.56
0.95
1.99
2.01
6.02
2.00
7.5 7.0 6.5 6.0 5.5 5.0 4.5 4.0 3.5 3.0 2.5 2.0 1.5 1.0 0.5
f1 (ppm)
B
159.00
118.33
117.42
114.55
114.35
113.03
76.21
75.78
59.37
58.97
46.11
45.34
43.77
43.19
23.57
23.09
170 160 150 140 130 120 110 100 90 80 70 60 50 40 30 20 10
f1 (ppm)

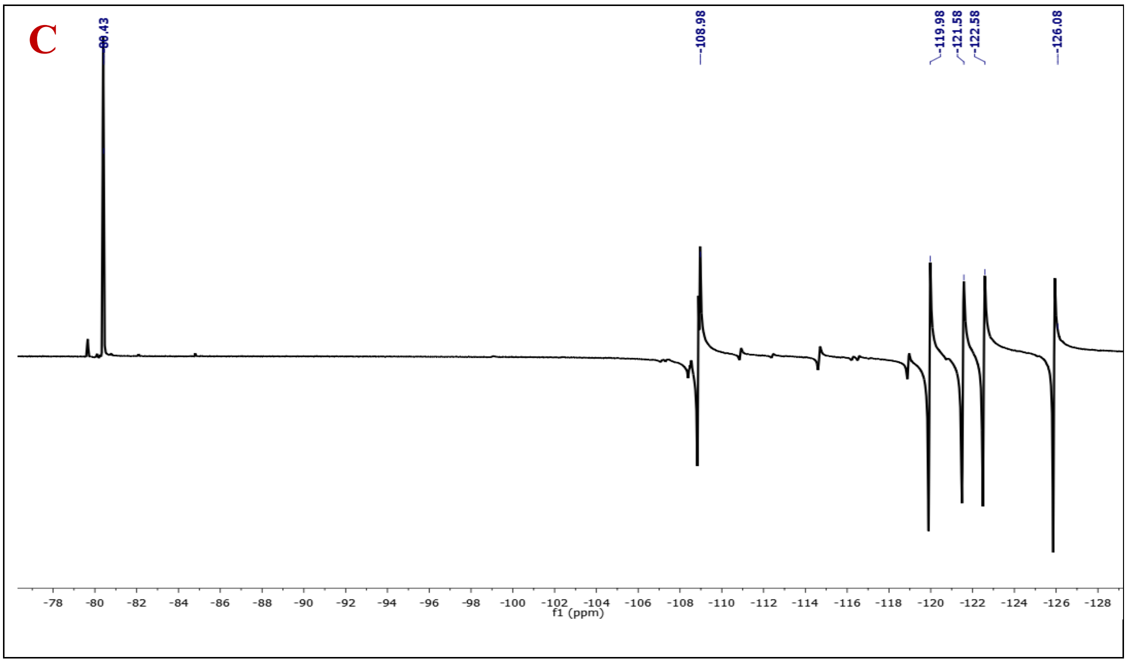

Figure S1. ^{1}H (A), ^{13}C (B) and F^{19} NMR(C) spectra of *N*-(3-(dimethylamino)propyl)-1,1,2,2,3,3,4,4,5,5,6,6,6-tridecafluorohexane-1-sulfonamide

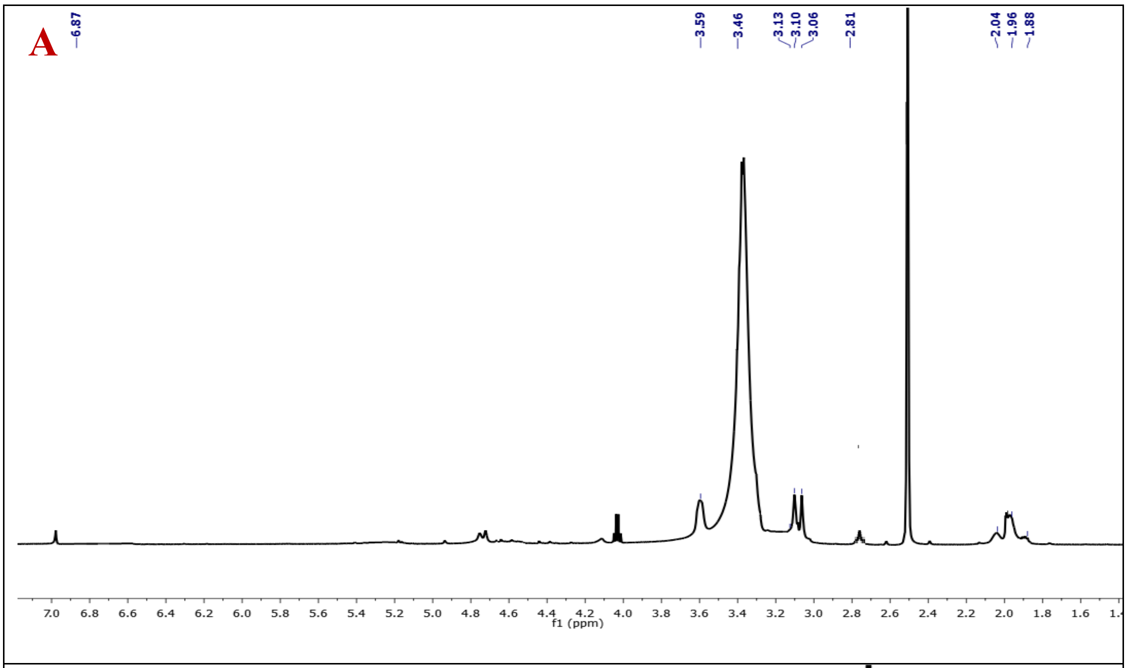
A
6.87
3.59
3.46
3.13
3.10
3.06
2.81
2.04
1.96
1.88
7.0 6.8 6.6 6.4 6.2 6.0 5.8 5.6 5.4 5.2 5.0 4.8 4.6 4.4 4.2 4.0 3.8 3.6 3.4 3.2 3.0 2.8 2.6 2.4 2.2 2.0 1.8 1.6
f1 (ppm)

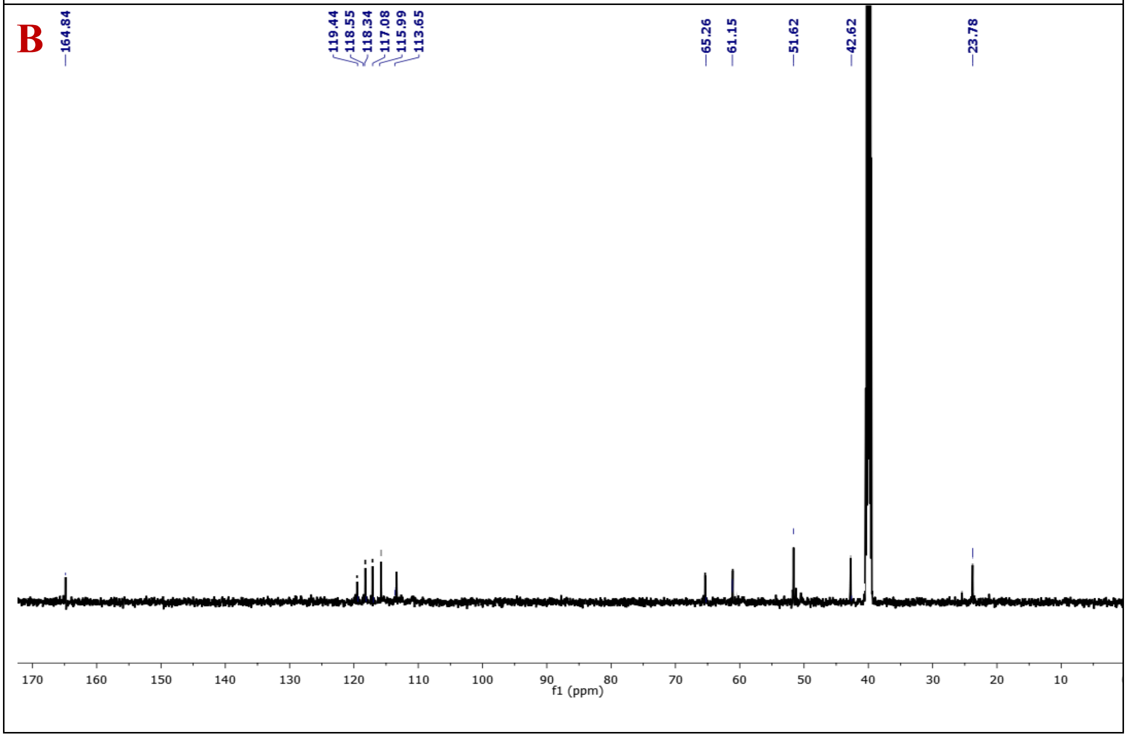
B
164.84
119.44
118.55
118.34
117.08
115.99
113.65
65.26
61.15
51.62
42.62
23.78
170 160 150 140 130 120 110 100 90 80 70 60 50 40 30 20 10
f1 (ppm)

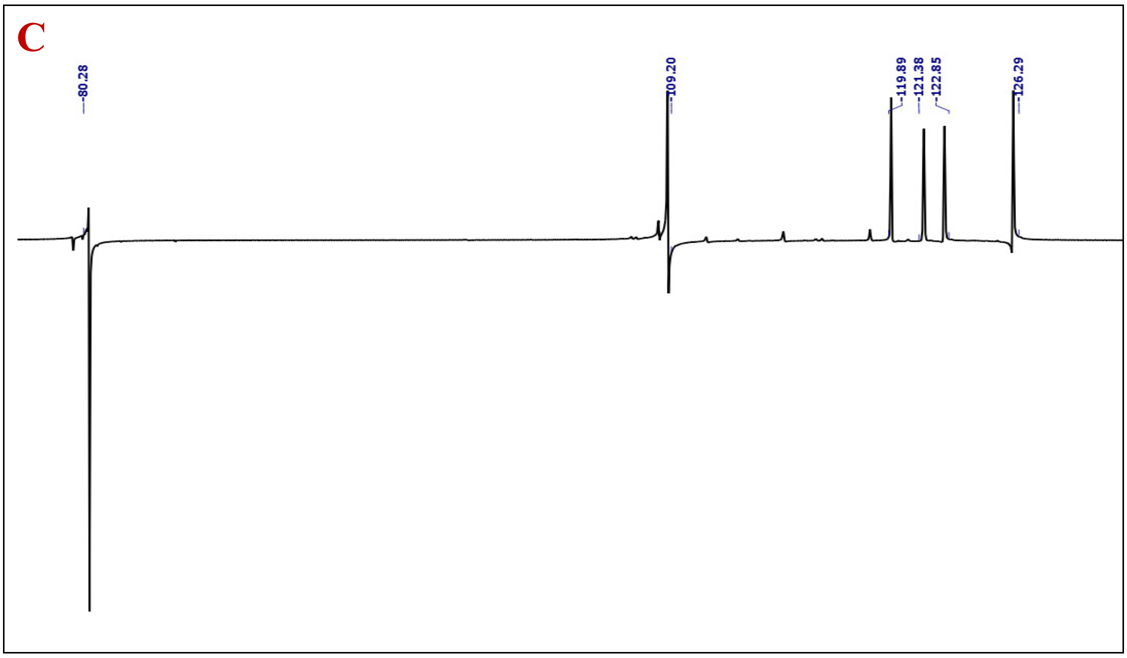

Figure S2. ^{1}H (A), ^{13}C (B) and F^{19} NMR(C) spectra of *N^{1},N^{1},N^{4},N^{4}*-tetramethyl-*N^{1},N^{4}*-bis(3-((perfluorohexyl)sulfonamido)propyl)butane-1,4-diaminium iodide (**3a, FSG6-2**).

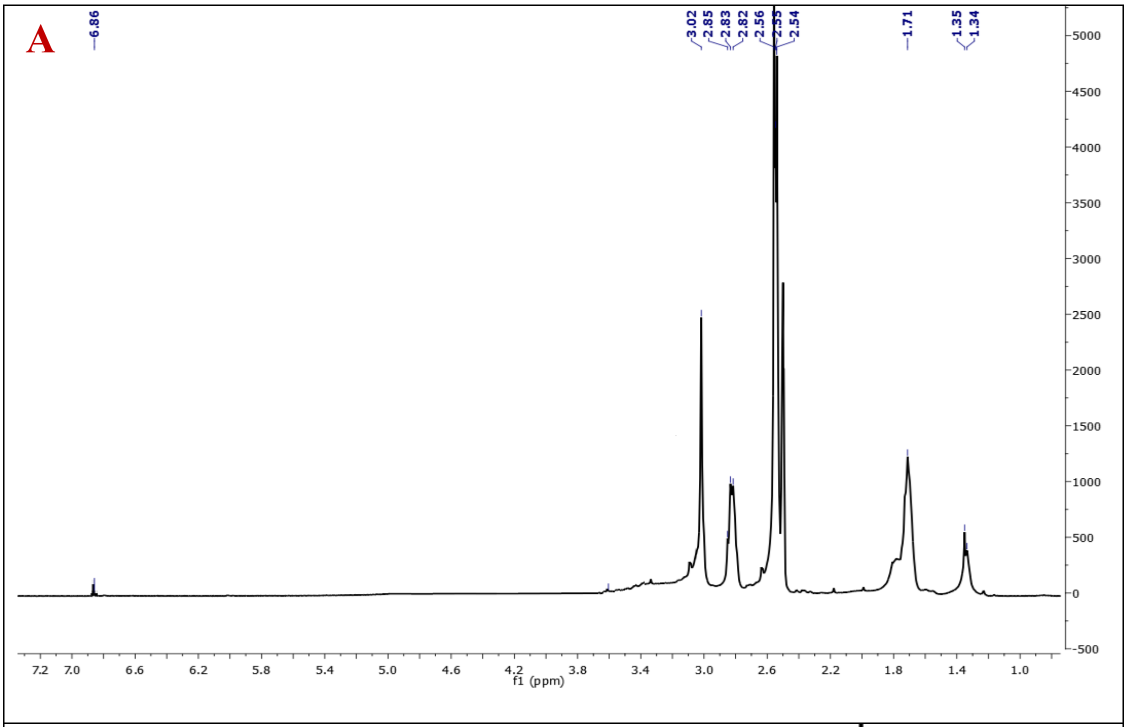
A
6.86
3.02
2.85
2.83
2.82
2.56
2.55
2.54
1.71
1.35
1.34
f1 (ppm)
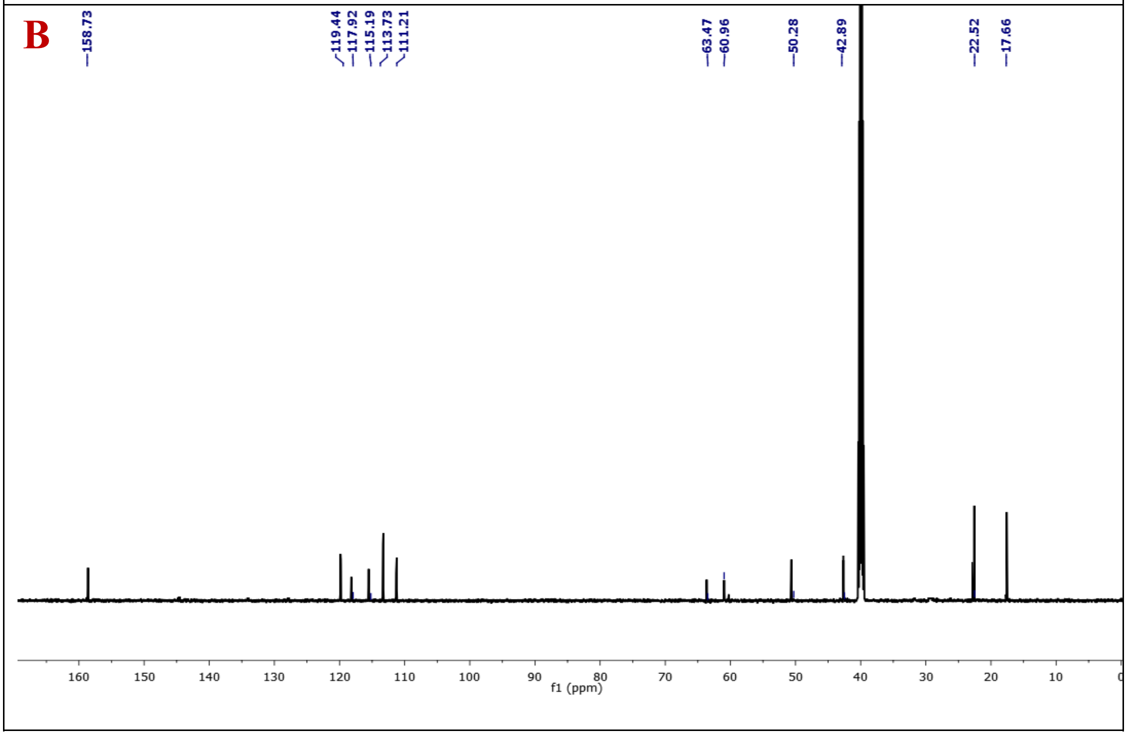
B
158.73
119.44
117.92
115.19
113.73
111.21
63.47
60.96
50.28
42.89
22.52
17.66
f1 (ppm)

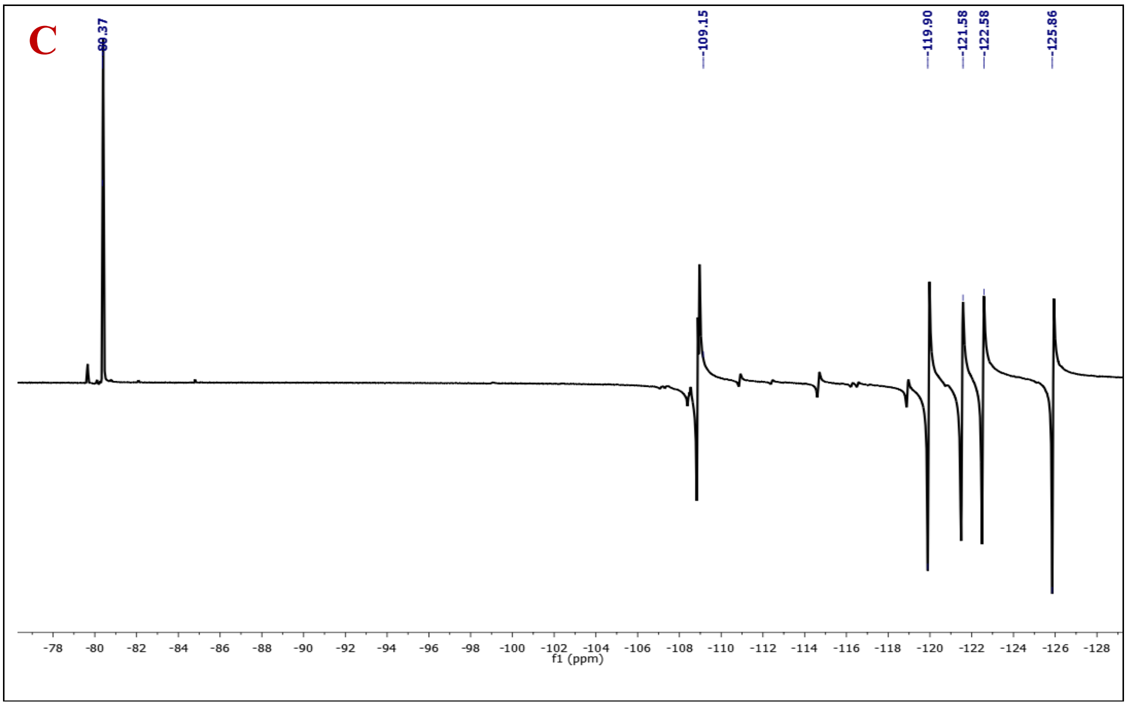

Figure S3. ^{1}H (A), ^{13}C (B) and F^{19} NMR(C) spectra of N^{1},N^{1},N^{4},N^{4}-tetramethyl-N^{1},N^{4}-bis(3-((perfluorohexyl)sulfonamido)propyl)butane-1,4-diaminium iodide (**3b, FSG6-4**)

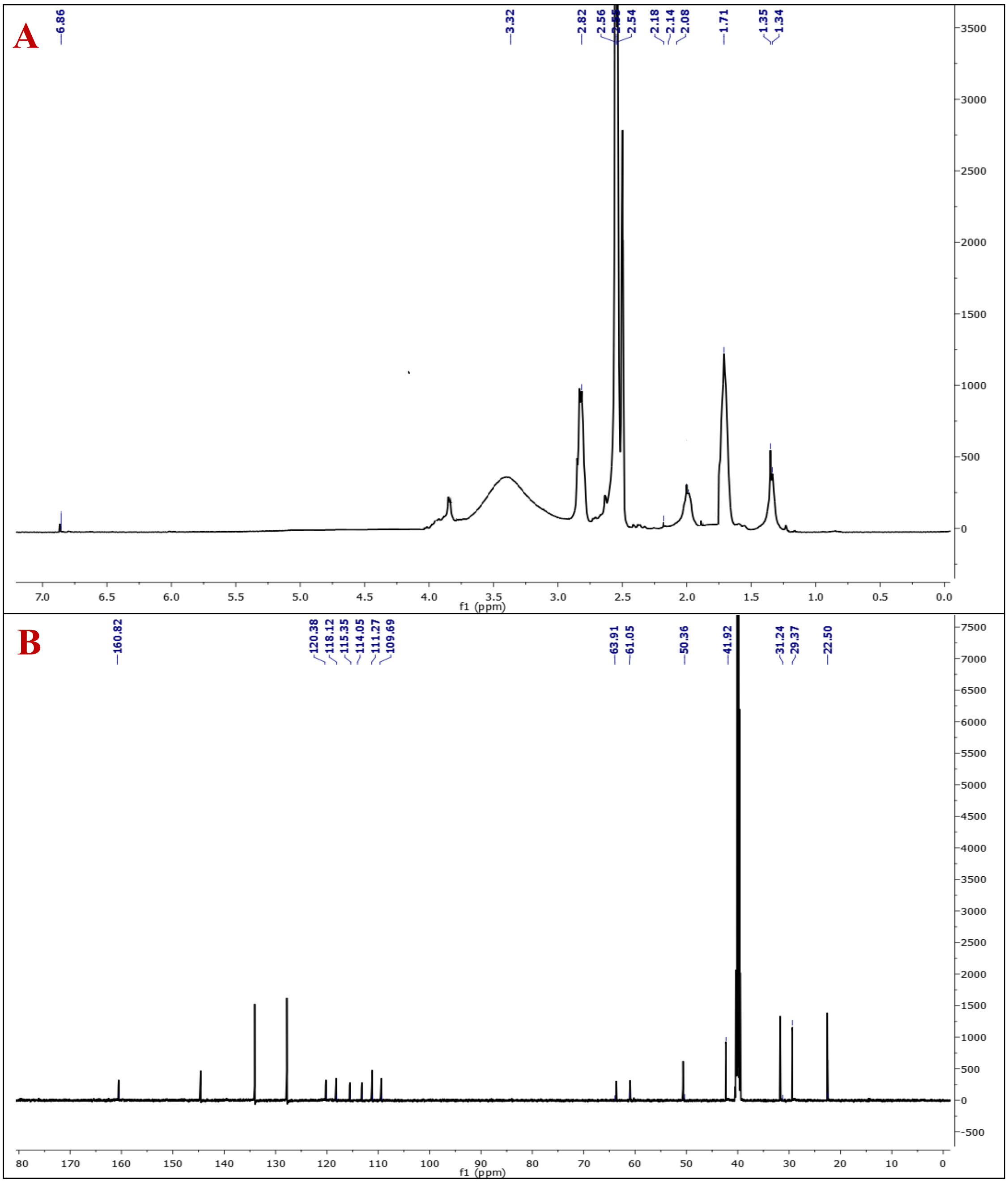
A
6.86
3.32
2.82
2.56
2.55
2.54
2.18
2.14
2.08
1.71
1.35
1.34
f1 (ppm)
B
160.82
120.38
118.12
115.35
114.05
111.27
109.69
63.91
61.05
50.36
41.92
31.24
29.37
22.50
f1 (ppm)

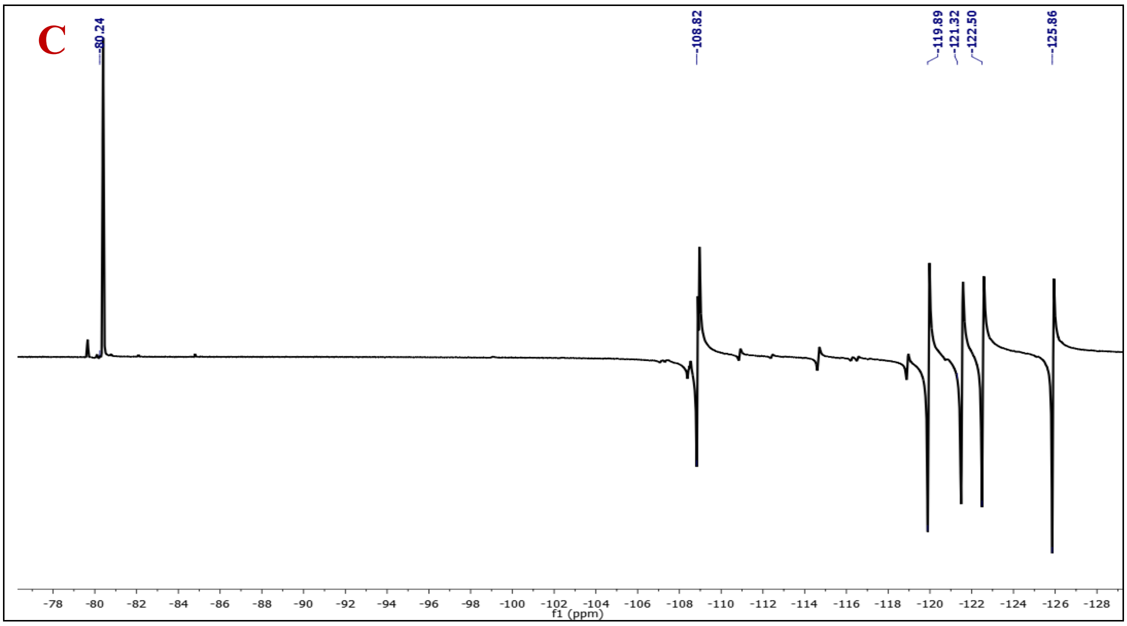

Figure S4. ^{1}H (A), ^{13}C (B) and F^{19} NMR(C) spectra of N^{1},N^{1},N^{6},N^{6}-tetramethyl-N^{1},N^{6}-bis(3-((perfluorohexyl)sulfonamido)propyl)hexane-1,6-diaminium iodide (**3c, FSG6-6**).
